# Supplementary material for: Study on pyroptosis-related genes Casp8, Gsdmd and Trem2 in mice with cerebral infarction
Source: PeerJ. 2024 Feb 9;12:e16818. doi: 10.7717/peerj.16818 (PMC10860548; doi:10.7717/peerj.16818)
Supplement: Supplemental Information 1 [file peerj-12-16818-s001.zip › Raw data/Bioinformatics analysis/figures/Figure 1/volcano.pdf]

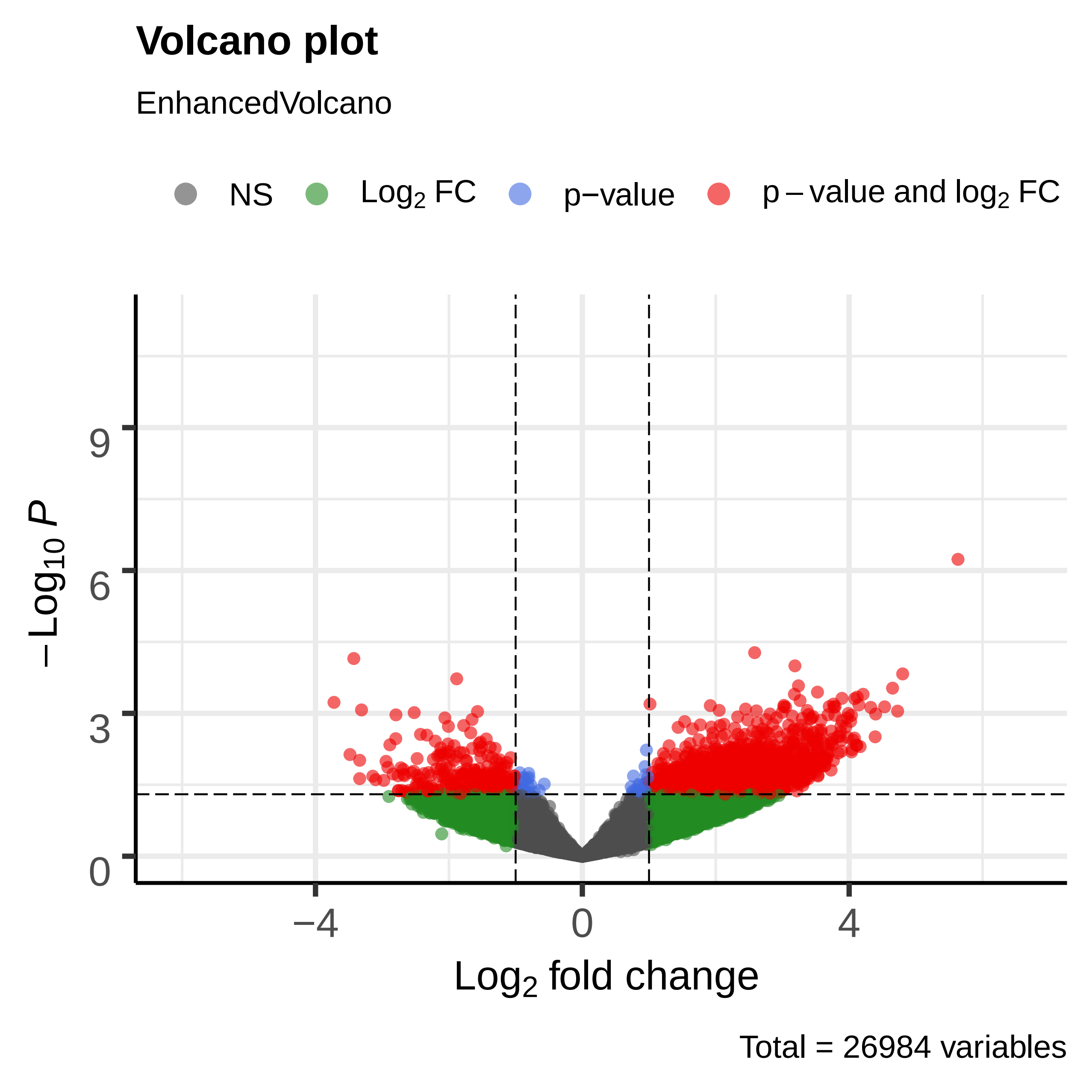
Volcano plot
EnhancedVolcano
NS
Log2 FC
p−value
p − value and log2 FC
9
6
3
0
−Log10 P
−4
0
4
Log2 fold change
Total = 26984 variables
