# Supplementary material for: Study on pyroptosis-related genes Casp8, Gsdmd and Trem2 in mice with cerebral infarction
Source: PeerJ. 2024 Feb 9;12:e16818. doi: 10.7717/peerj.16818 (PMC10860548; doi:10.7717/peerj.16818)
Supplement: Supplemental Information 1 [file peerj-12-16818-s001.zip › Raw data/Bioinformatics analysis/figures/Figure 3/GOenrich.pdf]

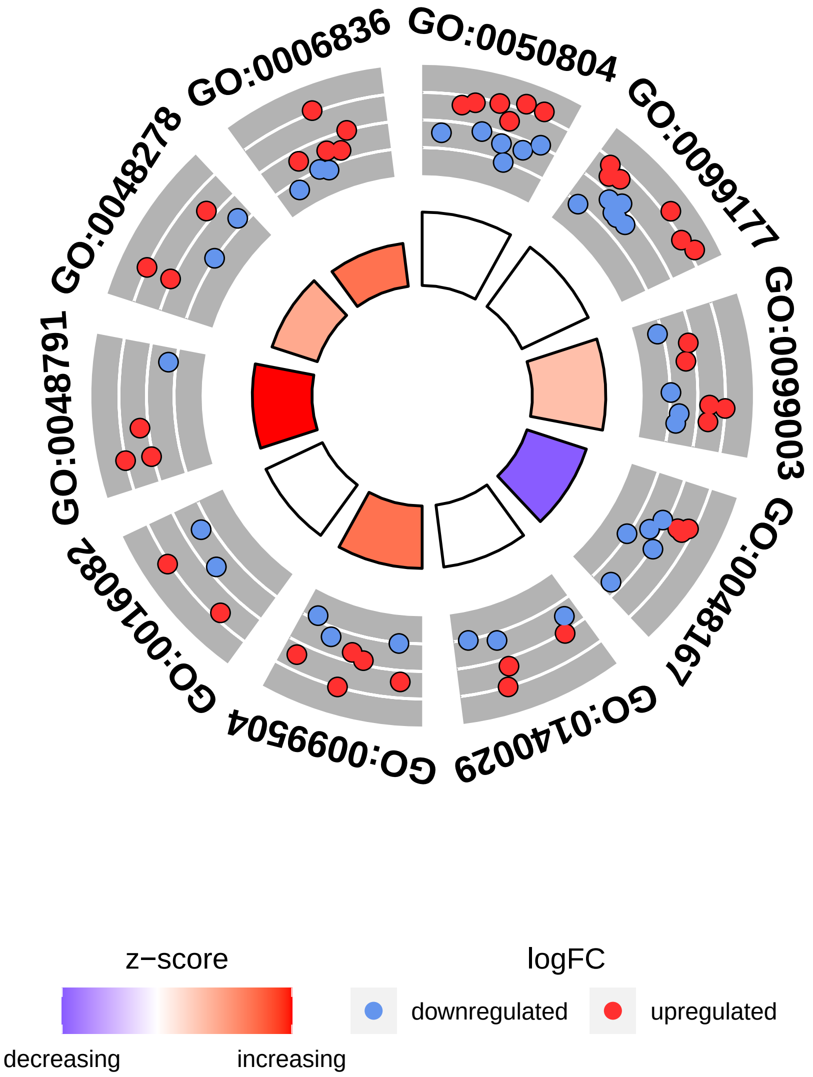

ID	Description
GO:0050804	modulation of chemical synaptic transmission
GO:0099177	regulation of trans−synaptic signaling
GO:0099003	vesicle−mediated transport in synapse
GO:0048167	regulation of synaptic plasticity
GO:0140029	exocytic process
GO:0099504	synaptic vesicle cycle
GO:0016082	synaptic vesicle priming
GO:0048791	calcium ion−regulated exocytosis of neurotransmitter
GO:0048278	vesicle docking
GO:0006836	neurotransmitter transport
