# Supplementary figures and images for: Study on pyroptosis-related genes Casp8, Gsdmd and Trem2 in mice with cerebral infarction
Source: PeerJ. 2024 Feb 9;12:e16818. doi: 10.7717/peerj.16818 (PMC10860548; doi:10.7717/peerj.16818)

# Cluster Dendrogram

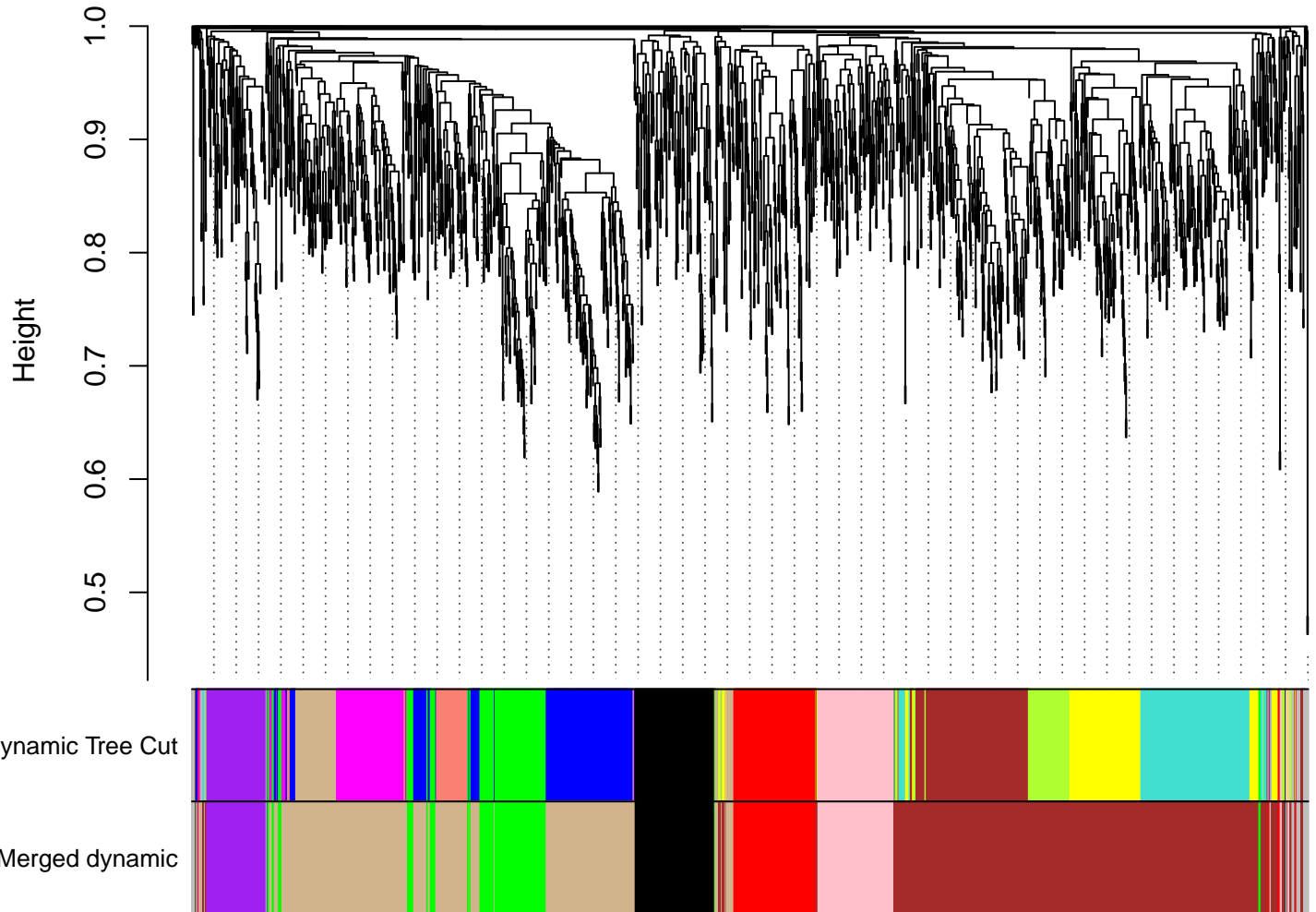

Supplement: Supplemental Information 1 [file peerj-12-16818-s001.zip › Raw data/Bioinformatics analysis/figures/Figure 2/geneTree.pdf]

**Module membership vs. gene significance**  
**cor=0.46, p=1.8e-07**

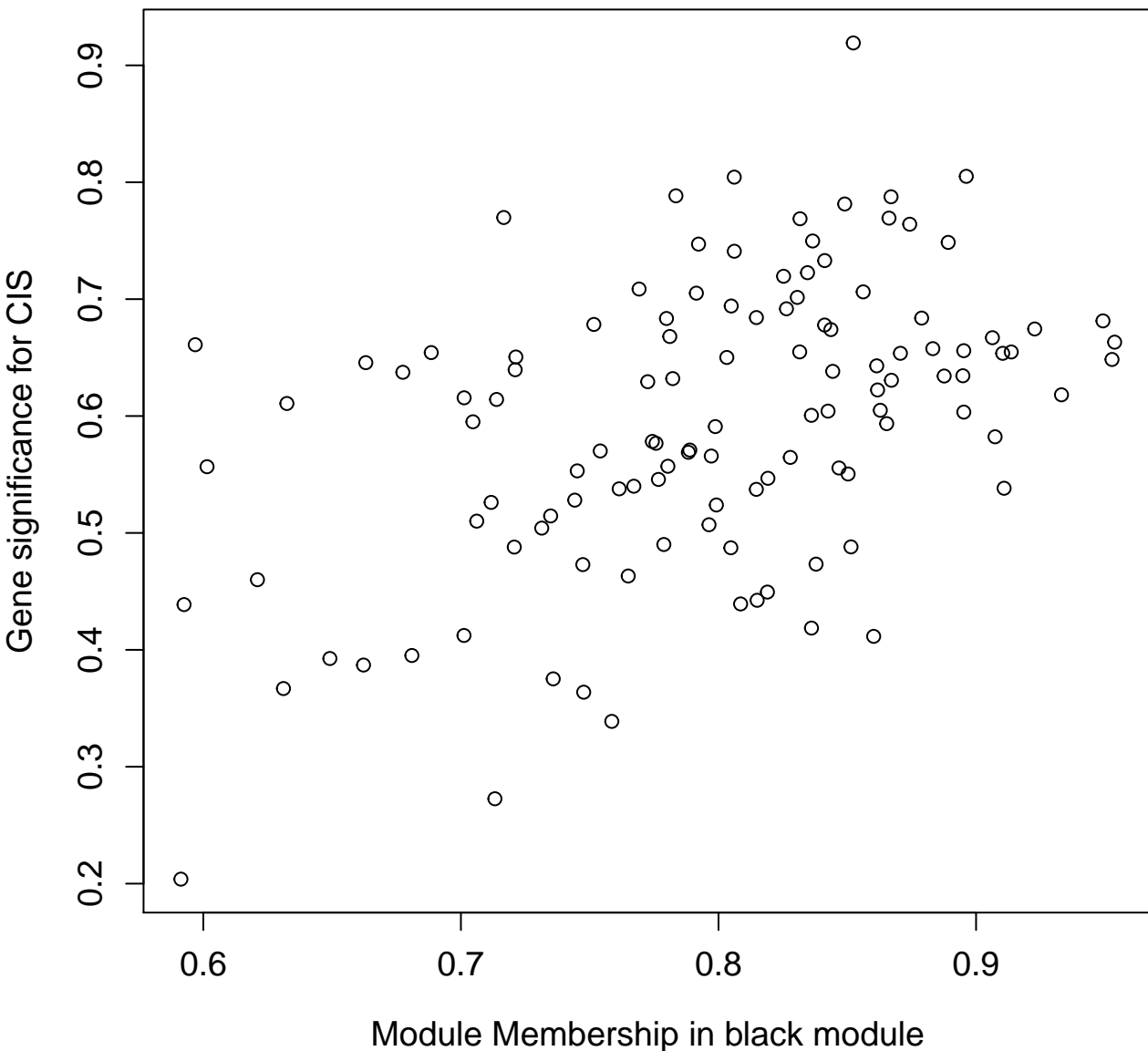

Supplement: Supplemental Information 1 [file peerj-12-16818-s001.zip › Raw data/Bioinformatics analysis/figures/Figure 2/MM-GS.pdf]

# Module-trait relationships

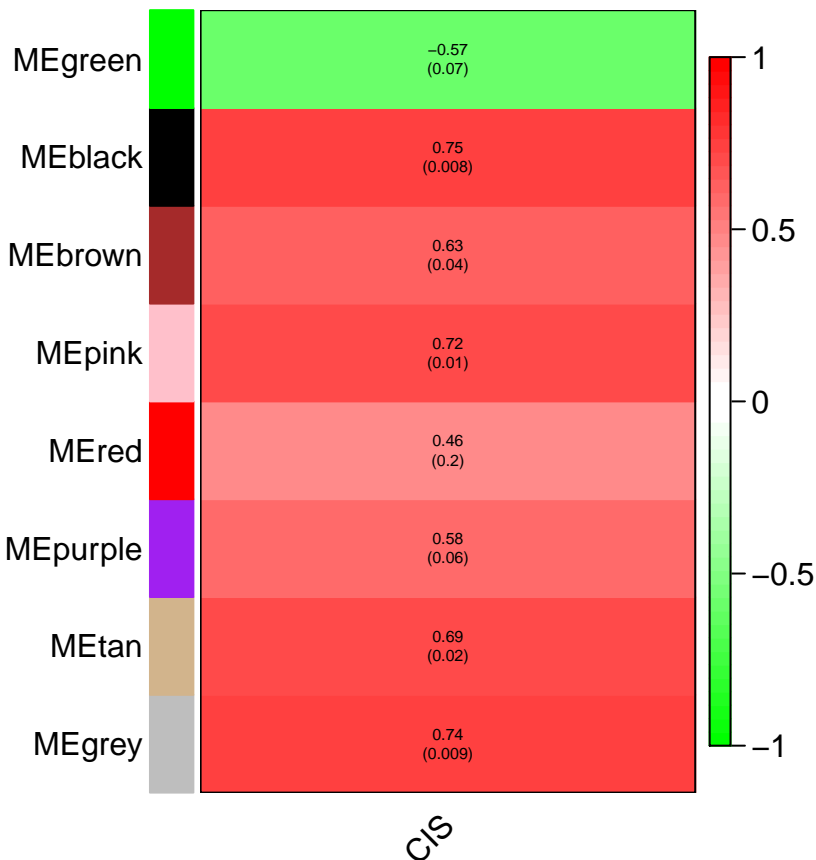

Supplement: Supplemental Information 1 [file peerj-12-16818-s001.zip › Raw data/Bioinformatics analysis/figures/Figure 2/mod-trait.pdf]

# Sample clustering to detect outliers

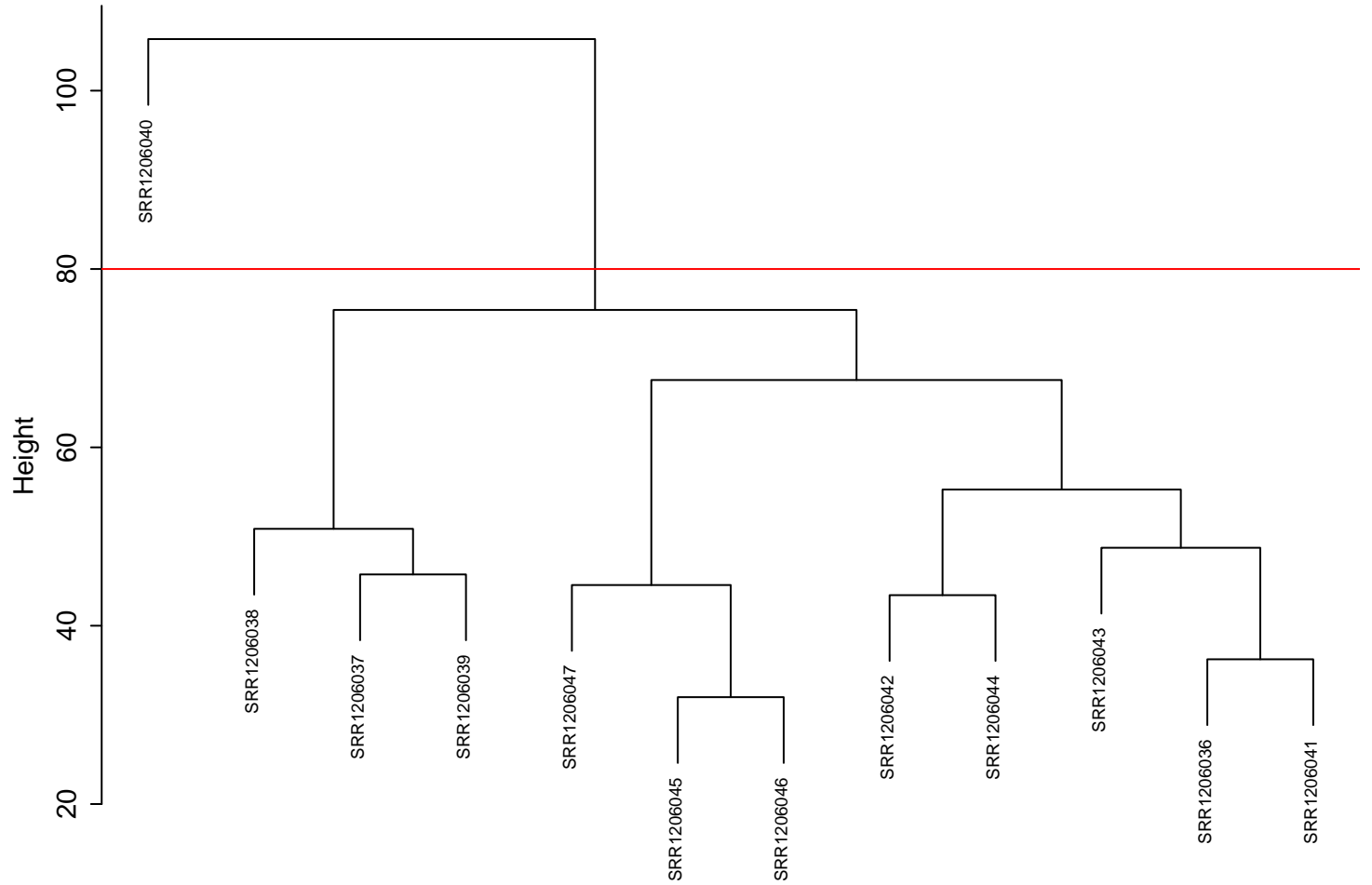

Supplement: Supplemental Information 1 [file peerj-12-16818-s001.zip › Raw data/Bioinformatics analysis/figures/Figure 2/sampleTree.pdf]

### Scale independence

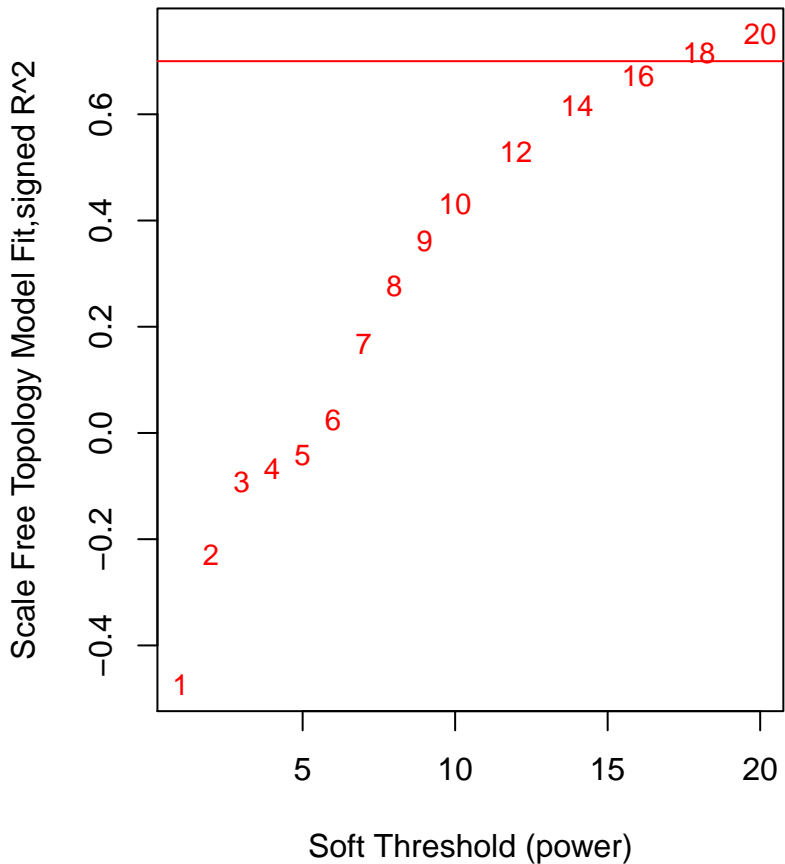

### Mean connectivity

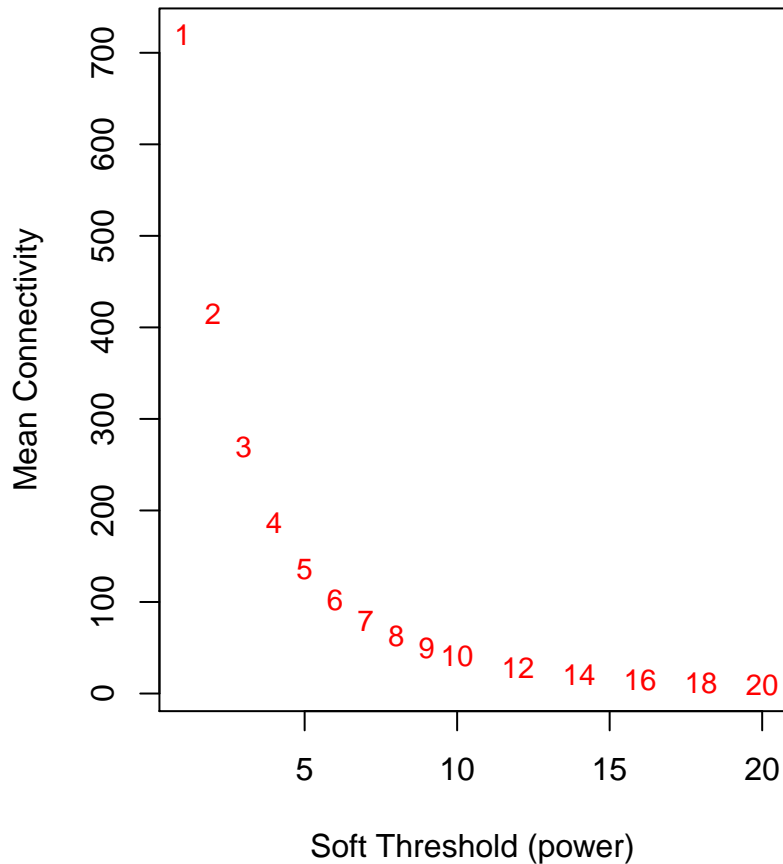

Supplement: Supplemental Information 1 [file peerj-12-16818-s001.zip › Raw data/Bioinformatics analysis/figures/Figure 2/sft.pdf]

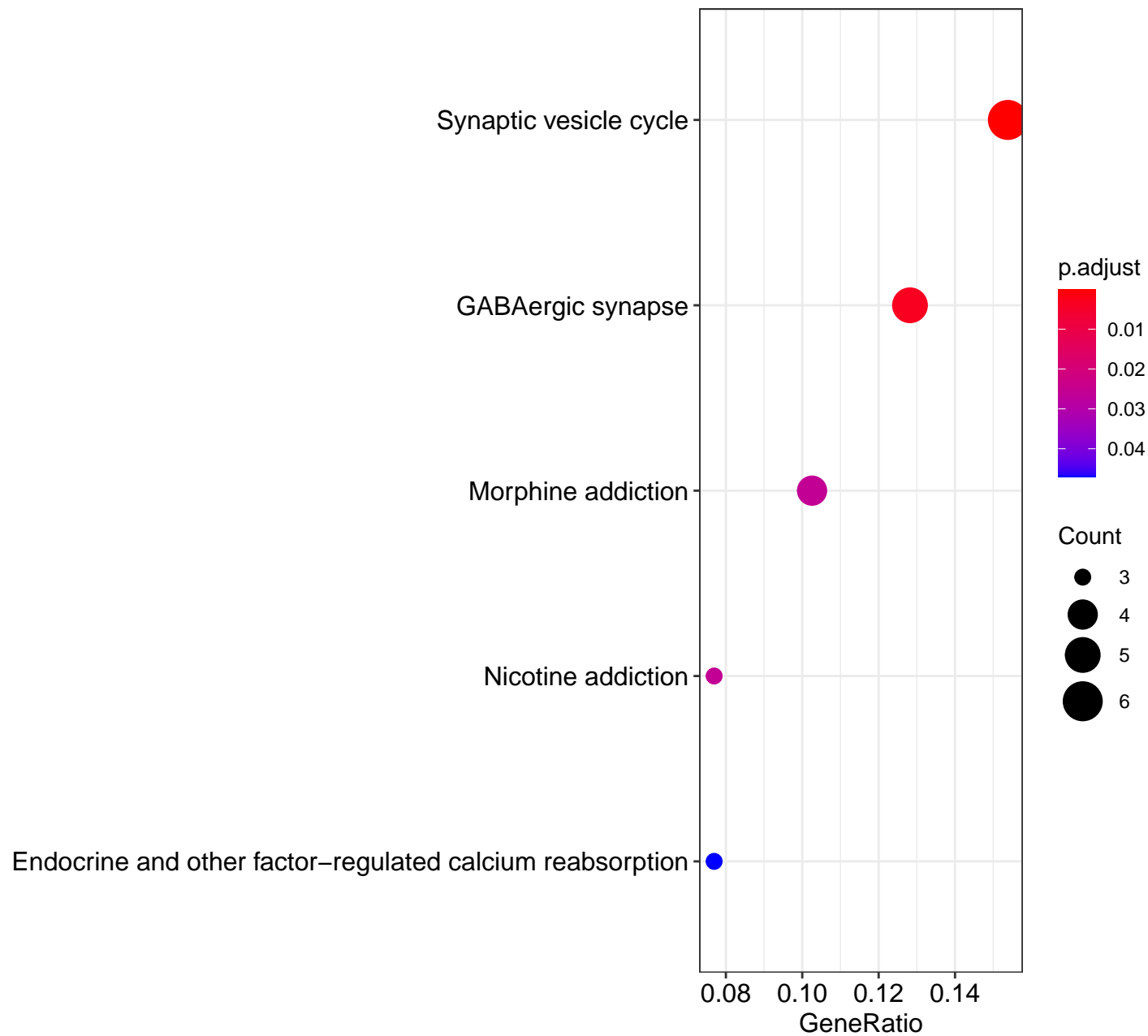

Supplement: Supplemental Information 1 [file peerj-12-16818-s001.zip › Raw data/Bioinformatics analysis/figures/Figure 3/kegg.pdf]

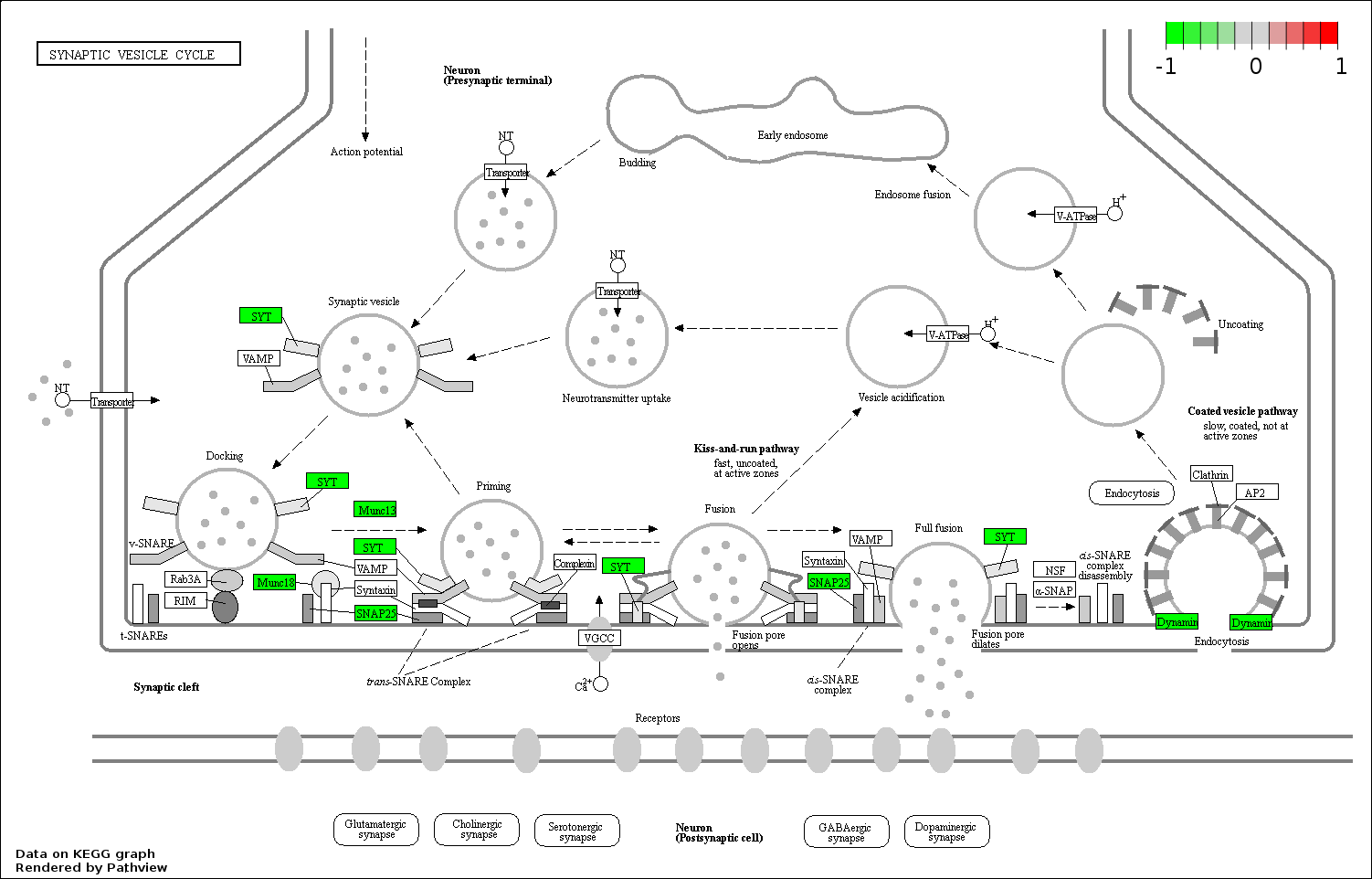

Supplement: Supplemental Information 1 [file peerj-12-16818-s001.zip › Raw data/Bioinformatics analysis/figures/Figure 4/hsa04721.pathview.png]

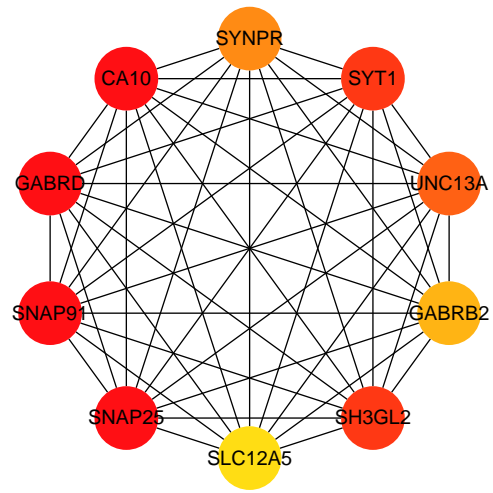

Supplement: Supplemental Information 1 [file peerj-12-16818-s001.zip › Raw data/Bioinformatics analysis/figures/Figure 4/MCC_top10.pdf]

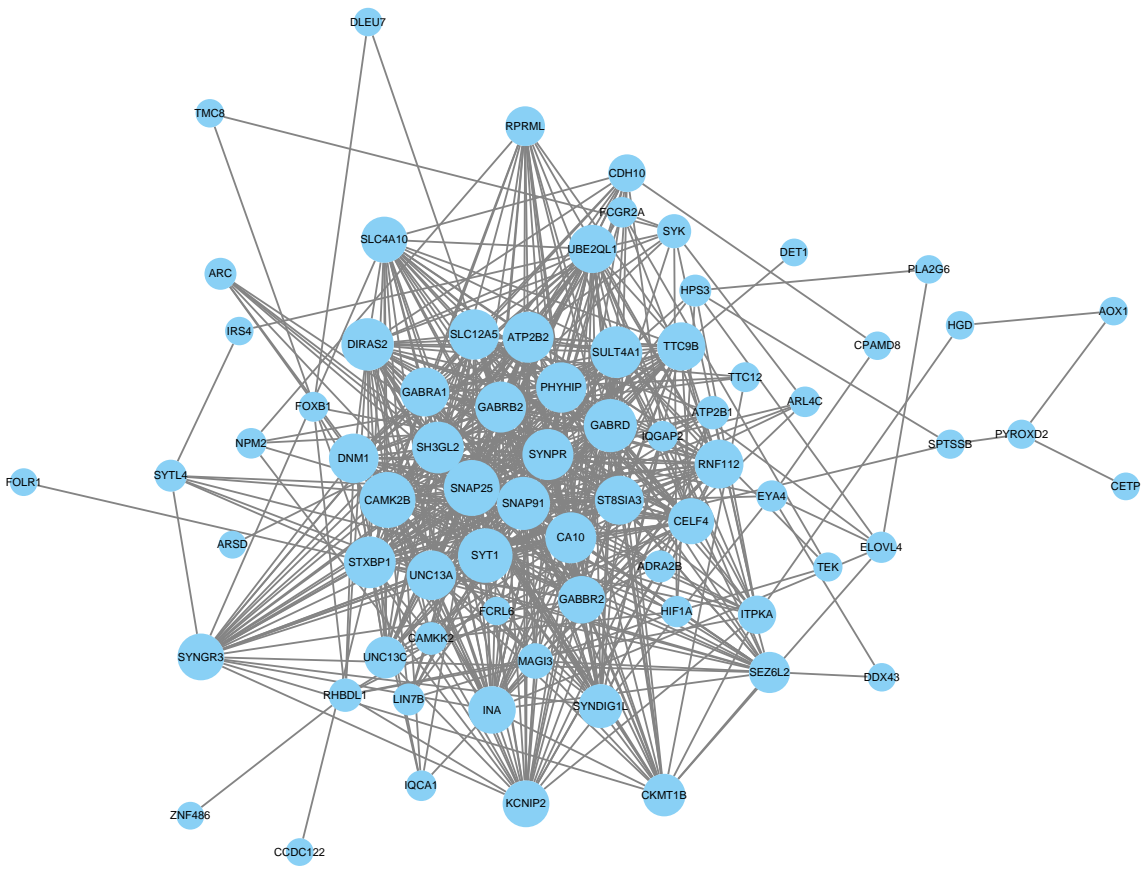

Supplement: Supplemental Information 1 [file peerj-12-16818-s001.zip › Raw data/Bioinformatics analysis/figures/Figure 4/PPI.pdf]

SNAP25 expression level

CIS

healthy

Tissue

CIS

healthy

0.27

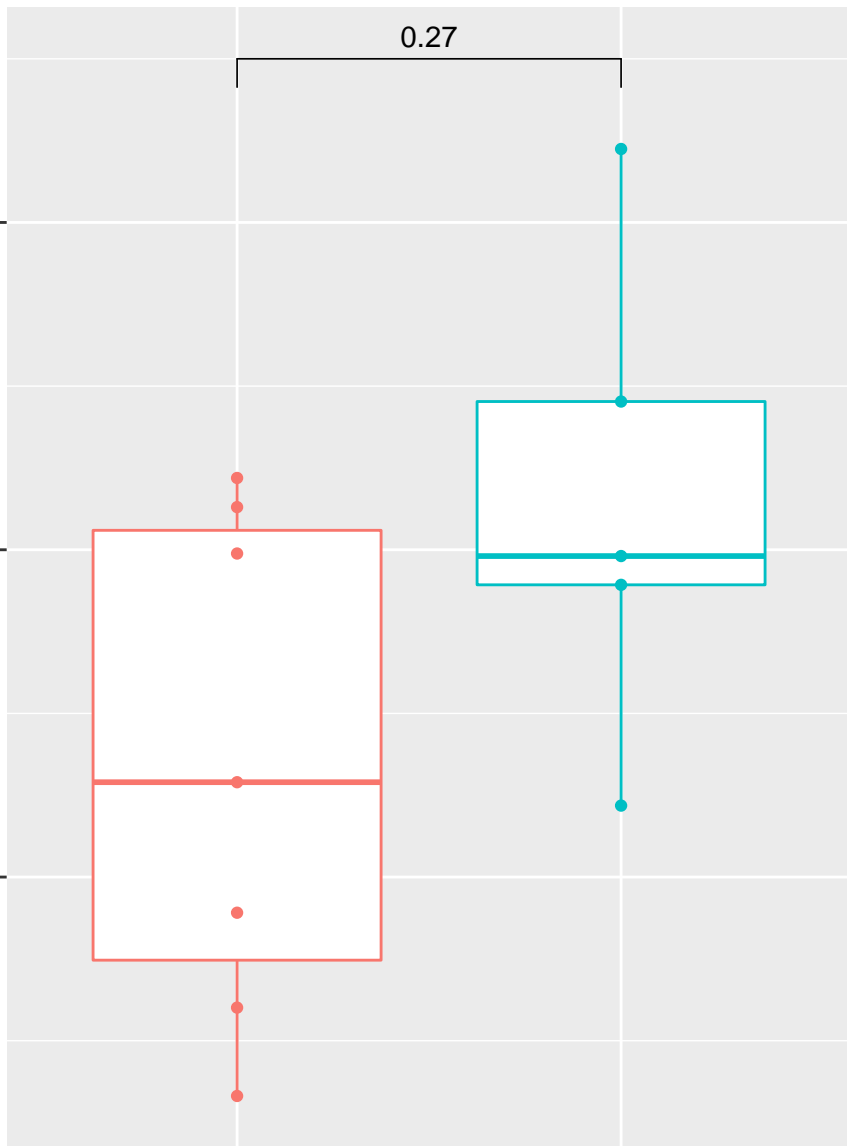

Supplement: Supplemental Information 1 [file peerj-12-16818-s001.zip › Raw data/Bioinformatics analysis/figures/Figure 5/SNAP25_box.pdf]

SYT1 expression level

0.018

10

8

6

CIS

healthy

Tissue

CIS

healthy

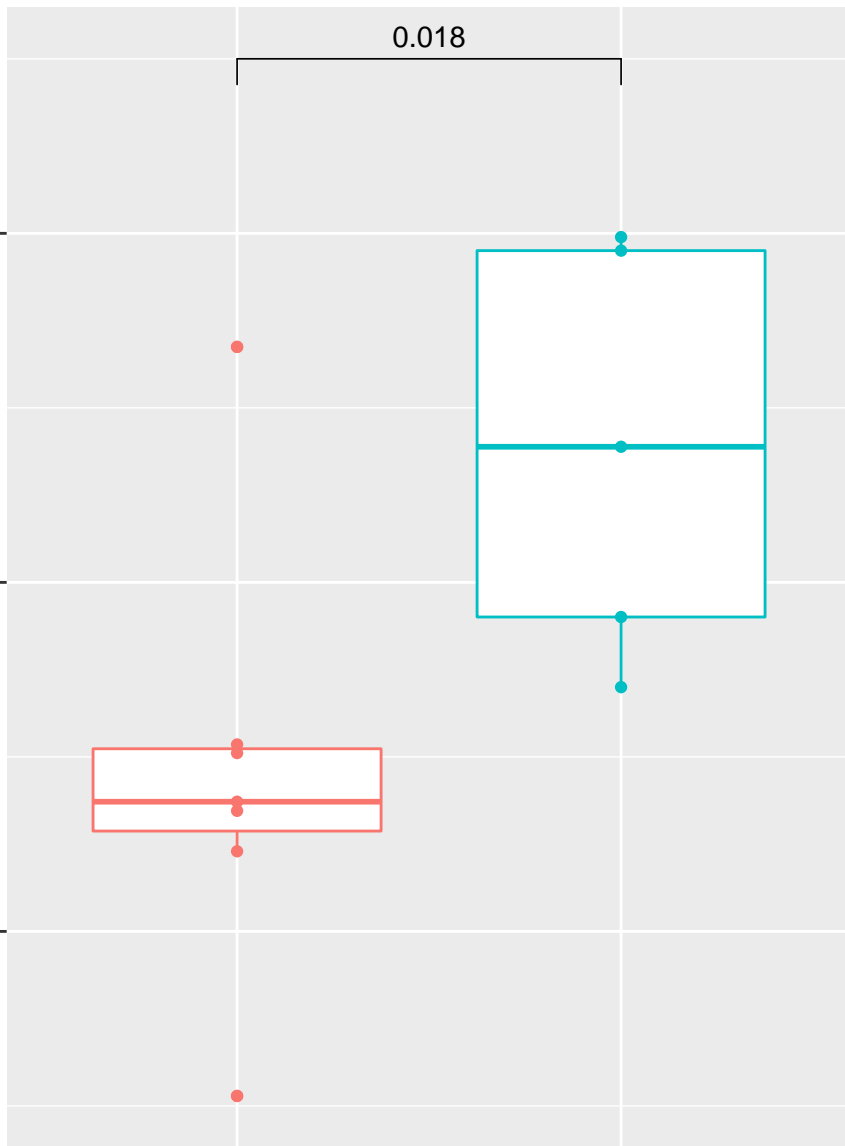

Supplement: Supplemental Information 1 [file peerj-12-16818-s001.zip › Raw data/Bioinformatics analysis/figures/Figure 5/SYT1_box.pdf]

UNC13A expression level

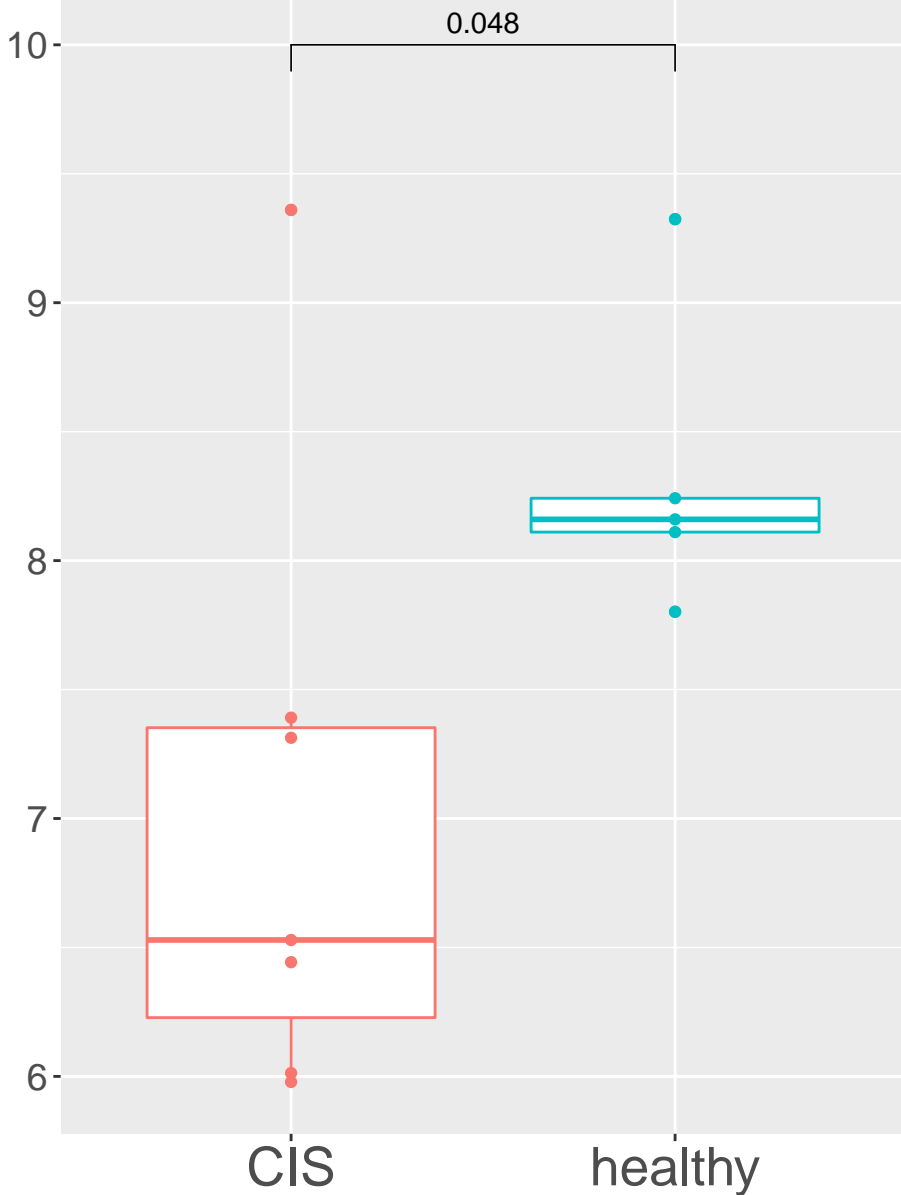

Tissue

CIS

healthy

Supplement: Supplemental Information 1 [file peerj-12-16818-s001.zip › Raw data/Bioinformatics analysis/figures/Figure 5/UNC13A_box.pdf]

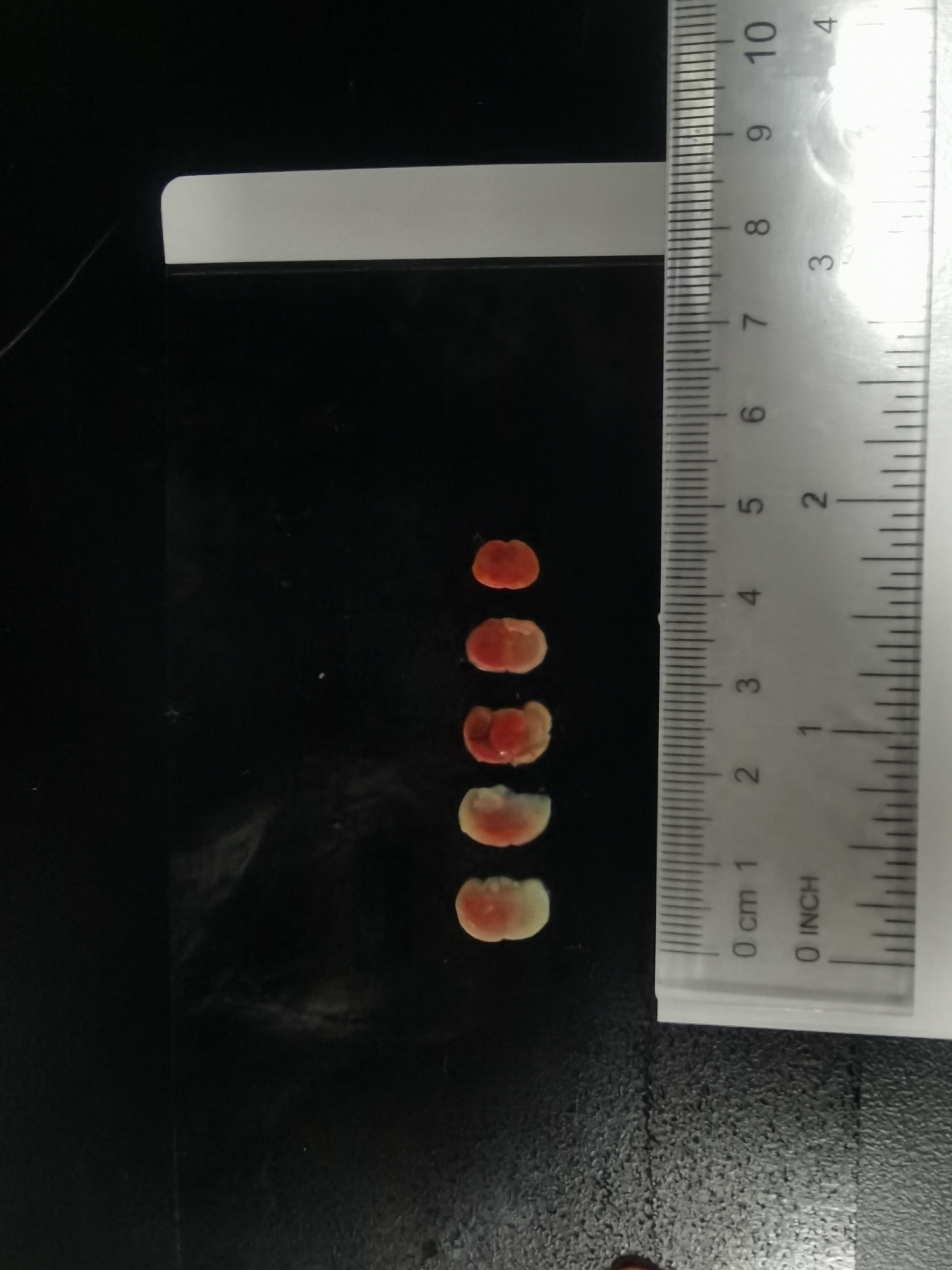

Supplement: Supplemental Information 1 [file peerj-12-16818-s001.zip › Raw data/TTC/MCAO.jpg]

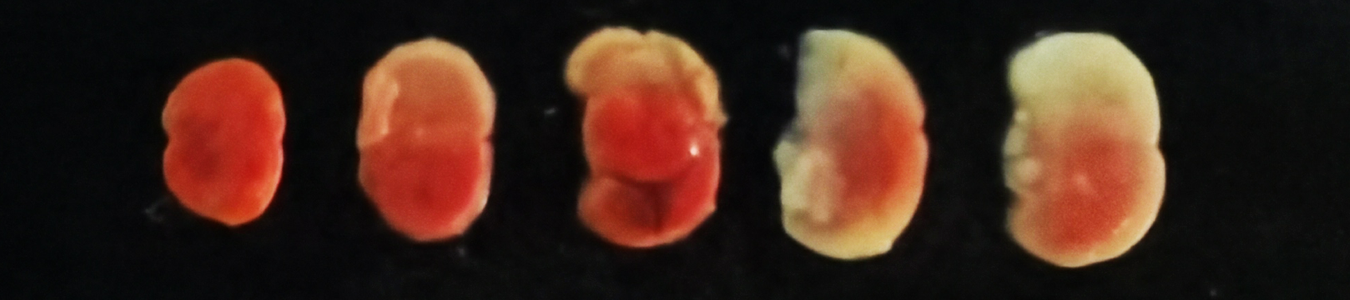

Supplement: Supplemental Information 1 [file peerj-12-16818-s001.zip › Raw data/TTC/MCAO-1.tif]

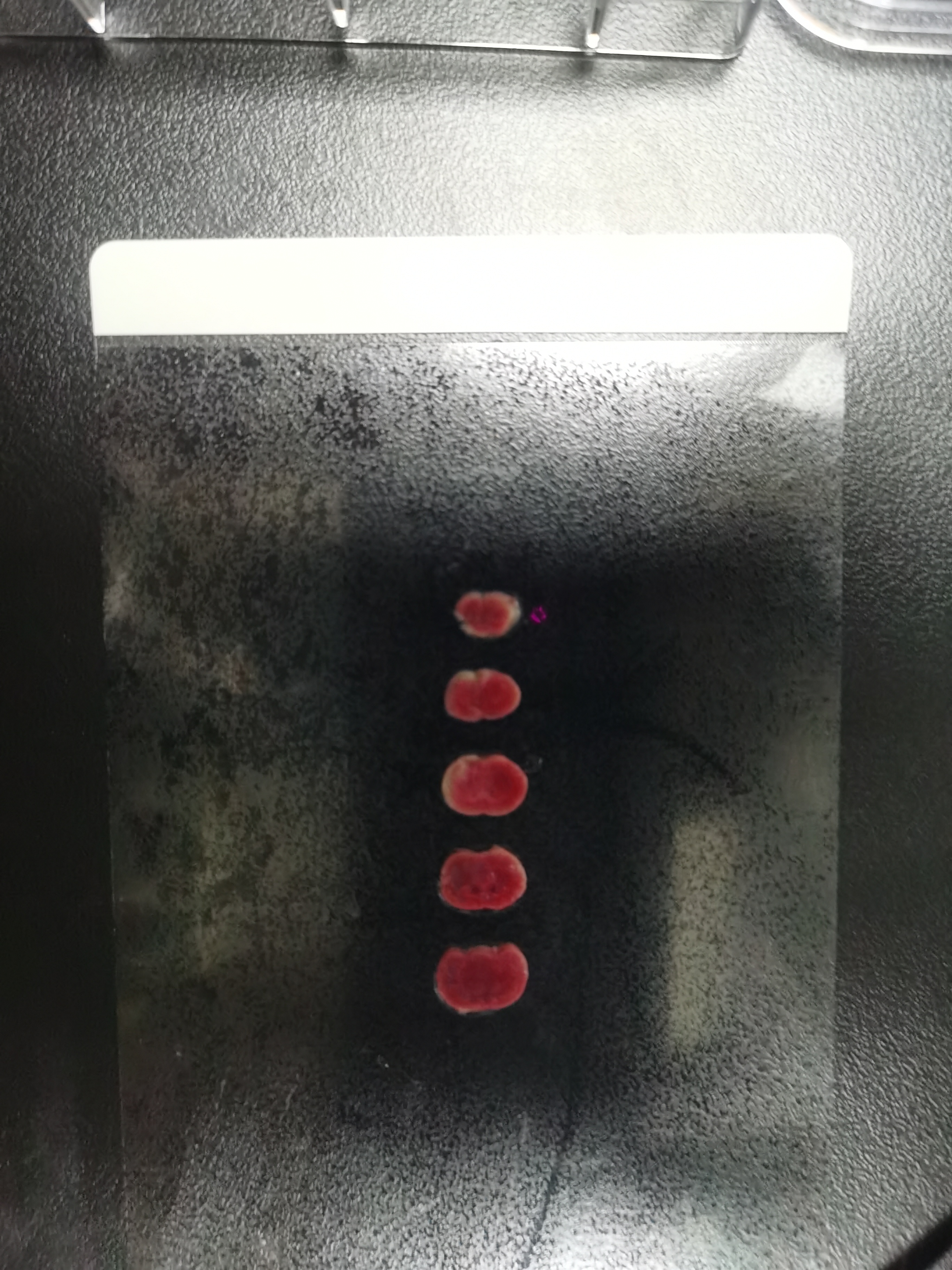

Supplement: Supplemental Information 1 [file peerj-12-16818-s001.zip › Raw data/TTC/sham.jpg]

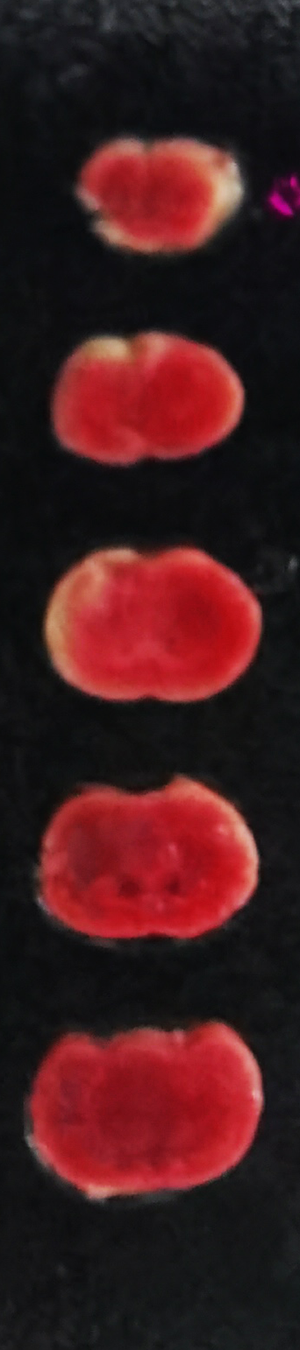

Supplement: Supplemental Information 1 [file peerj-12-16818-s001.zip › Raw data/TTC/sham-1.tif]

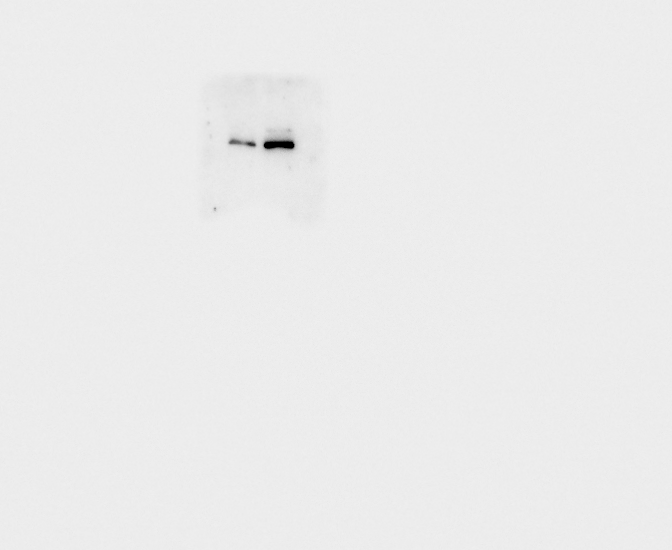

Supplement: Supplemental Information 1 [file peerj-12-16818-s001.zip › Raw data/WB/caspase8 1.tif]

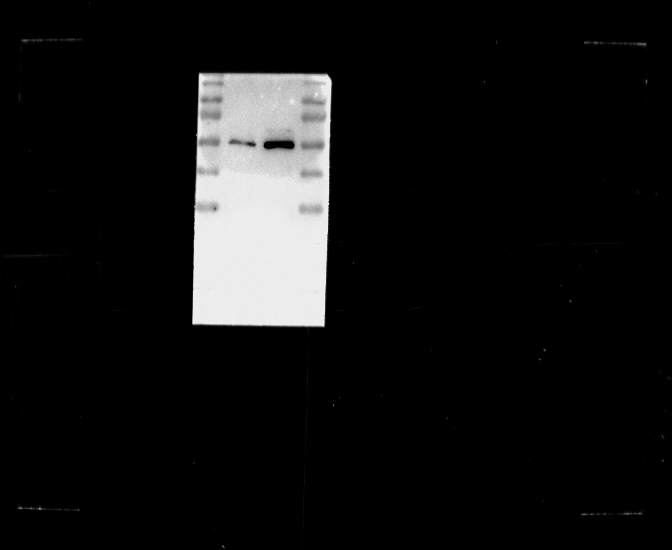

Supplement: Supplemental Information 1 [file peerj-12-16818-s001.zip › Raw data/WB/caspase8 2.tif]

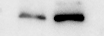

Supplement: Supplemental Information 1 [file peerj-12-16818-s001.zip › Raw data/WB/caspase8 3.tif]

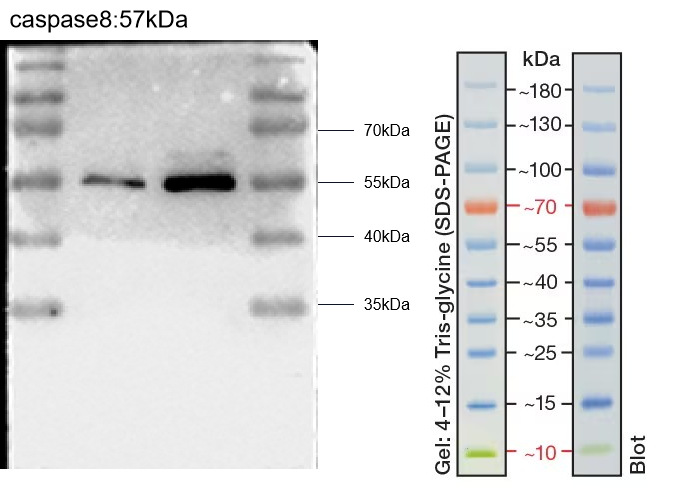

Supplement: Supplemental Information 1 [file peerj-12-16818-s001.zip › Raw data/WB/caspase8.tif]

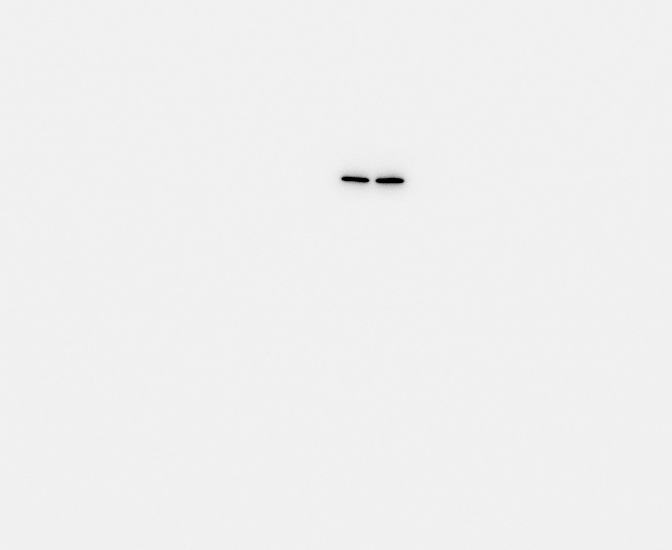

Supplement: Supplemental Information 1 [file peerj-12-16818-s001.zip › Raw data/WB/GAPDH 1.tif]

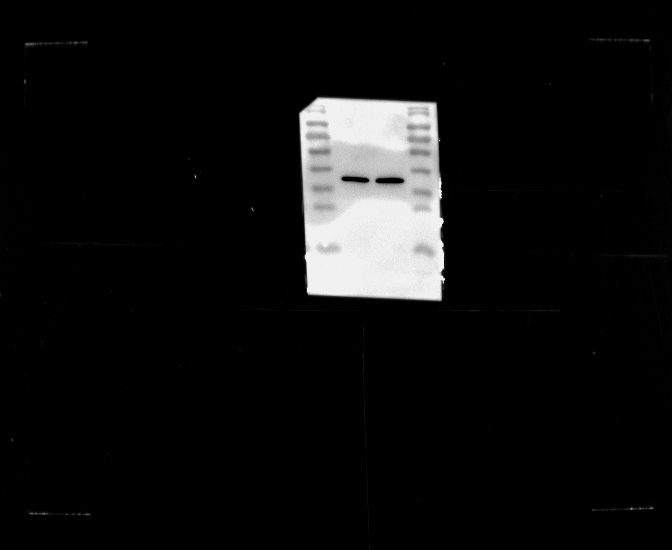

Supplement: Supplemental Information 1 [file peerj-12-16818-s001.zip › Raw data/WB/GAPDH 2.tif]

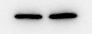

Supplement: Supplemental Information 1 [file peerj-12-16818-s001.zip › Raw data/WB/GAPDH 3.tif]

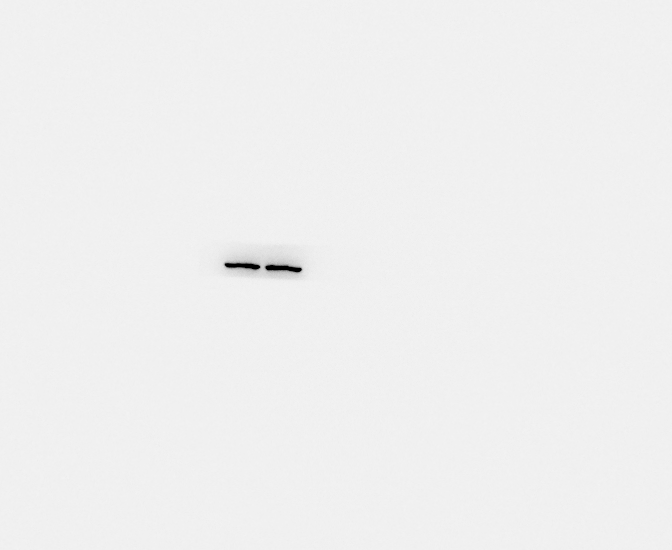

Supplement: Supplemental Information 1 [file peerj-12-16818-s001.zip › Raw data/WB/GAPDH 4.tif]

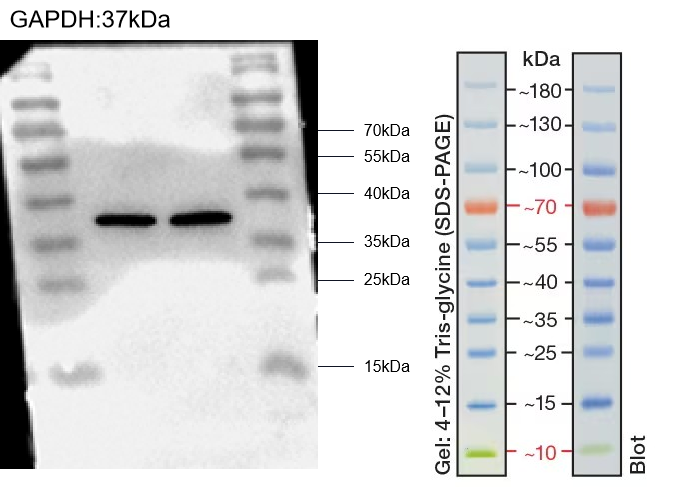

Supplement: Supplemental Information 1 [file peerj-12-16818-s001.zip › Raw data/WB/GAPDH.tif]

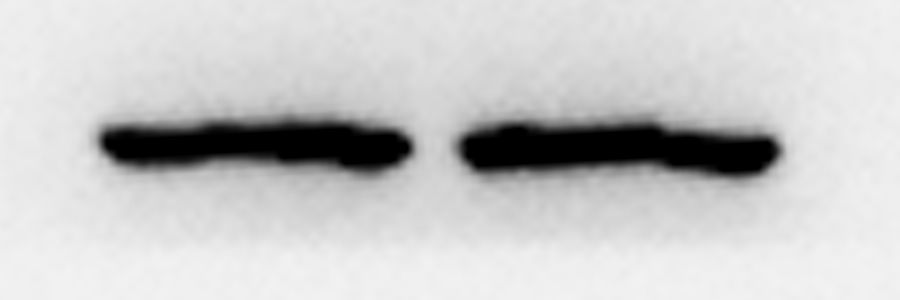

Supplement: Supplemental Information 1 [file peerj-12-16818-s001.zip › Raw data/WB/GAPDH-37kd.tif]

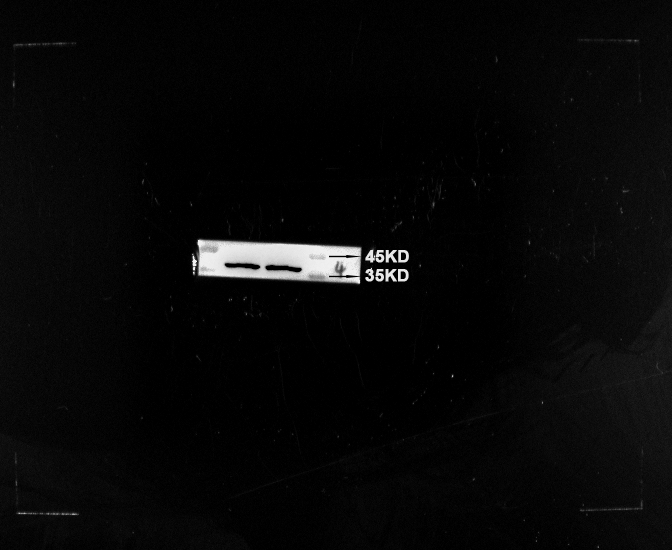

Supplement: Supplemental Information 1 [file peerj-12-16818-s001.zip › Raw data/WB/GAPDH-Marker.tif]

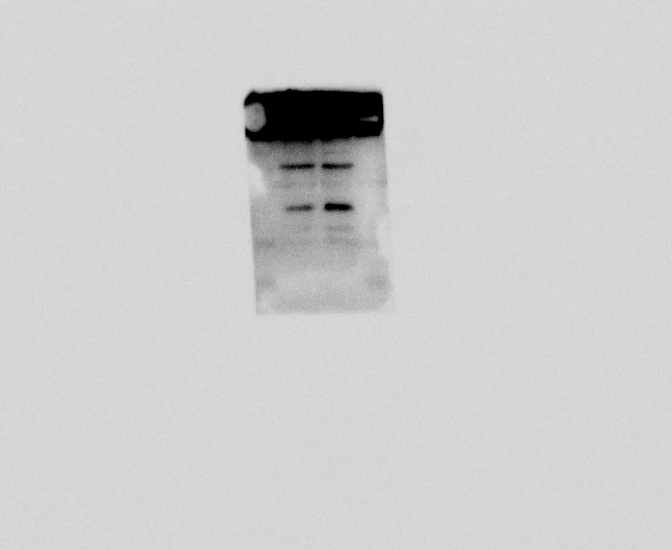

Supplement: Supplemental Information 1 [file peerj-12-16818-s001.zip › Raw data/WB/GSDMD 1.tif]

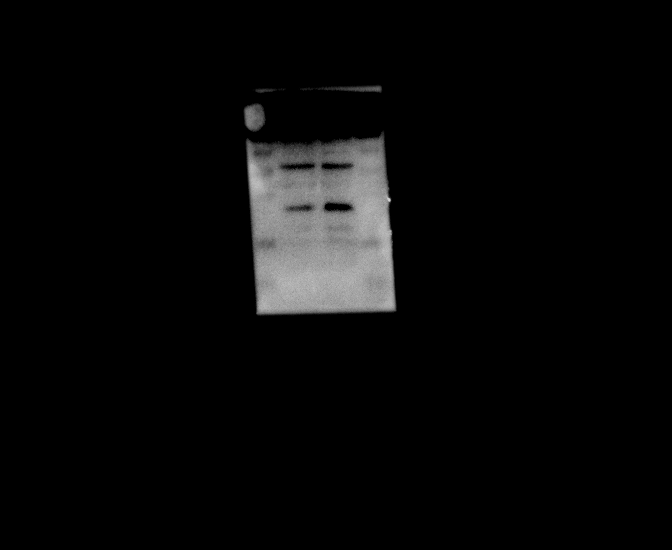

Supplement: Supplemental Information 1 [file peerj-12-16818-s001.zip › Raw data/WB/GSDMD 2.tif]

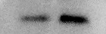

Supplement: Supplemental Information 1 [file peerj-12-16818-s001.zip › Raw data/WB/GSDMD 3.tif]

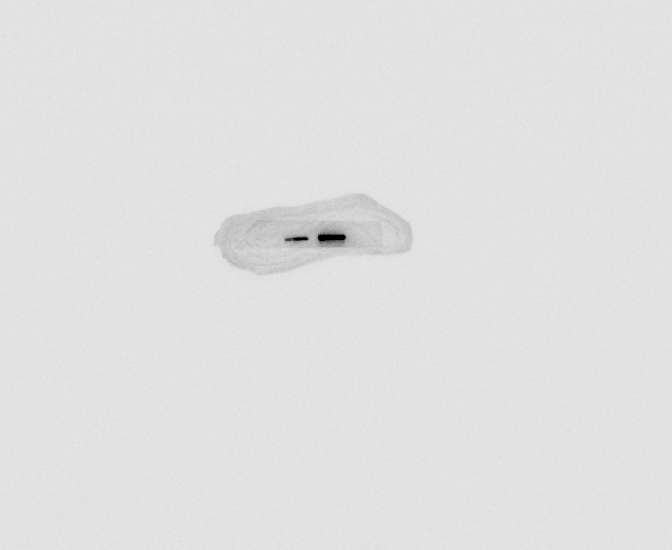

Supplement: Supplemental Information 1 [file peerj-12-16818-s001.zip › Raw data/WB/Gsdmd 4.tif]

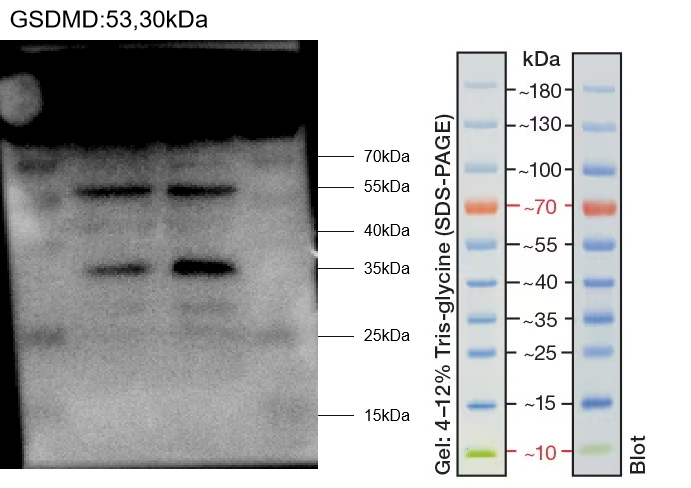

Supplement: Supplemental Information 1 [file peerj-12-16818-s001.zip › Raw data/WB/GSDMD.tif]

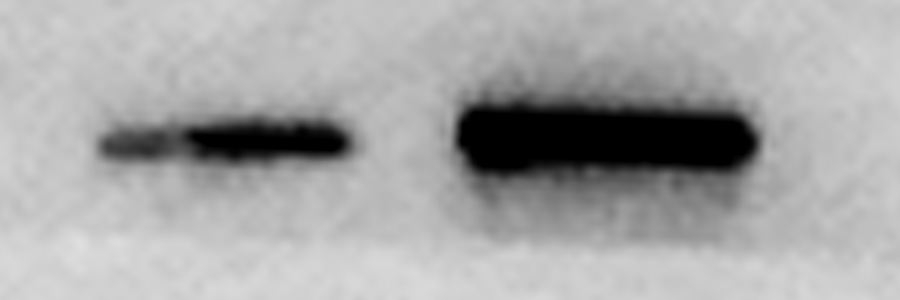

Supplement: Supplemental Information 1 [file peerj-12-16818-s001.zip › Raw data/WB/Gsdmd-53kd.tif]

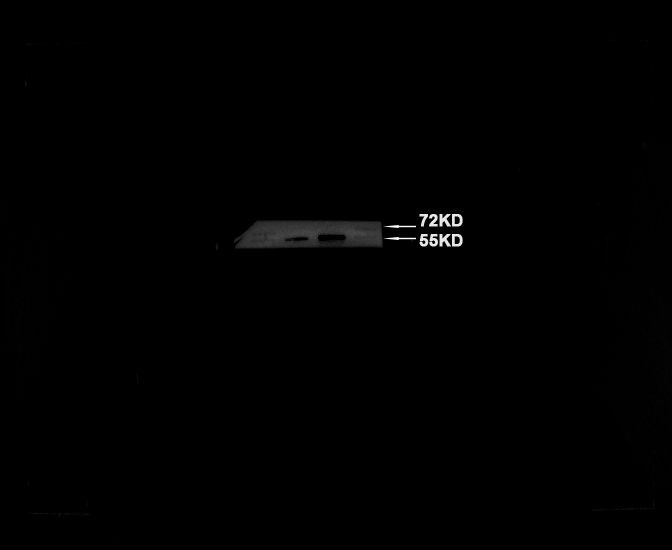

Supplement: Supplemental Information 1 [file peerj-12-16818-s001.zip › Raw data/WB/Gsdmd-Marker.tif]

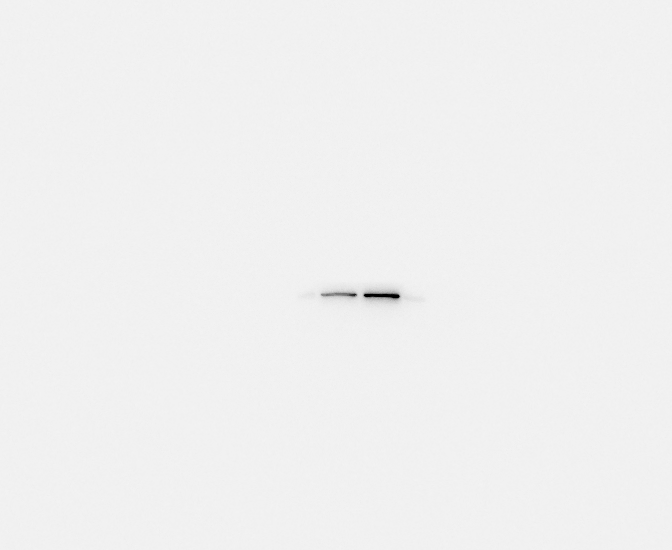

Supplement: Supplemental Information 1 [file peerj-12-16818-s001.zip › Raw data/WB/Gsp8.tif]

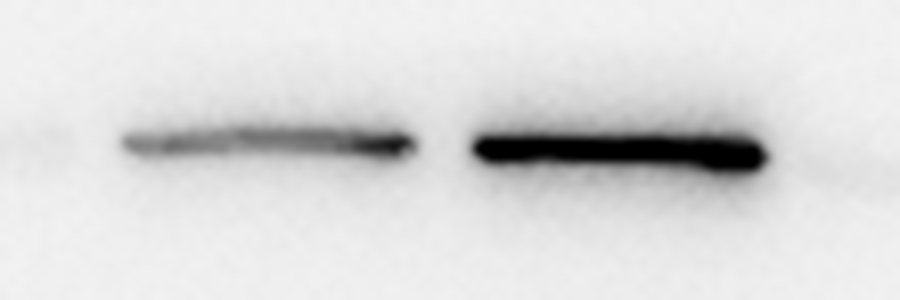

Supplement: Supplemental Information 1 [file peerj-12-16818-s001.zip › Raw data/WB/Gsp8-57kd.tif]

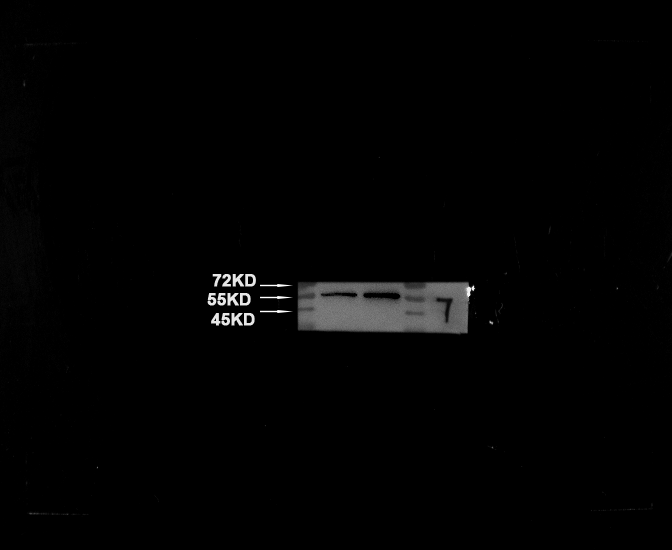

Supplement: Supplemental Information 1 [file peerj-12-16818-s001.zip › Raw data/WB/Gsp8-Marker.tif]

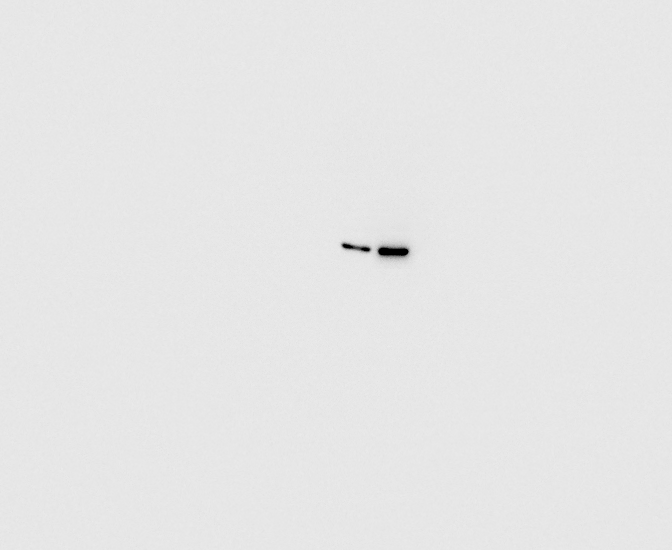

Supplement: Supplemental Information 1 [file peerj-12-16818-s001.zip › Raw data/WB/Trem2 1-1.tif]

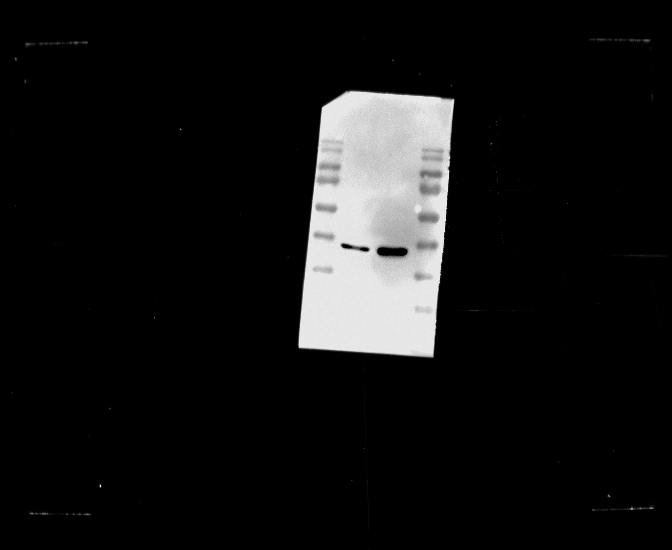

Supplement: Supplemental Information 1 [file peerj-12-16818-s001.zip › Raw data/WB/Trem2 2-1.tif]

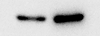

Supplement: Supplemental Information 1 [file peerj-12-16818-s001.zip › Raw data/WB/Trem2 3.tif]

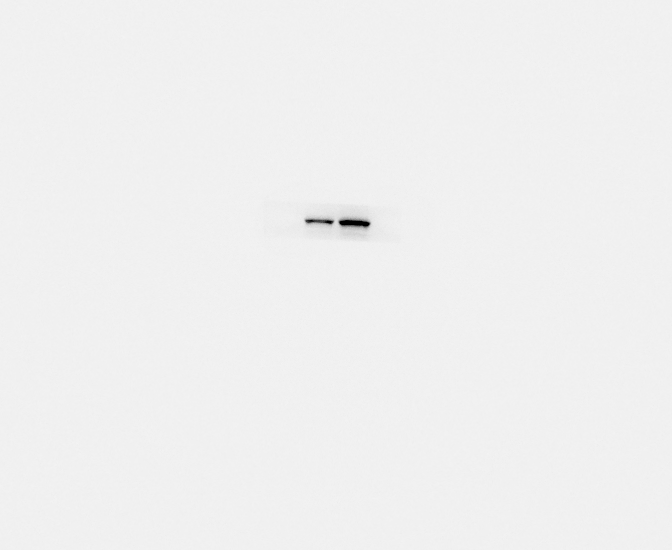

Supplement: Supplemental Information 1 [file peerj-12-16818-s001.zip › Raw data/WB/Trem2 4.tif]

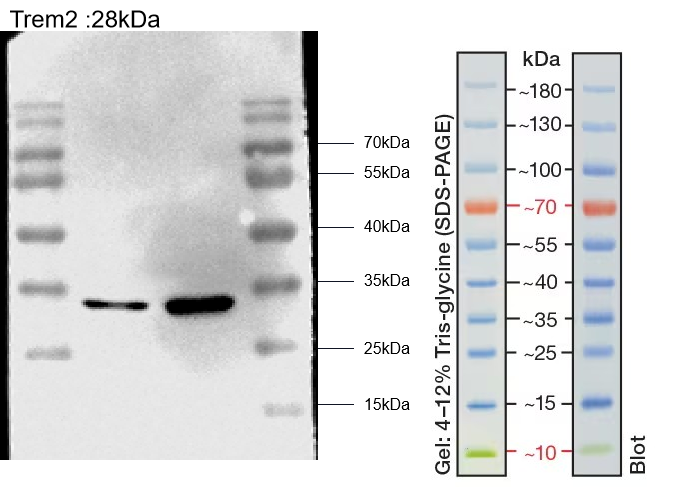

Supplement: Supplemental Information 1 [file peerj-12-16818-s001.zip › Raw data/WB/Trem2.tif]

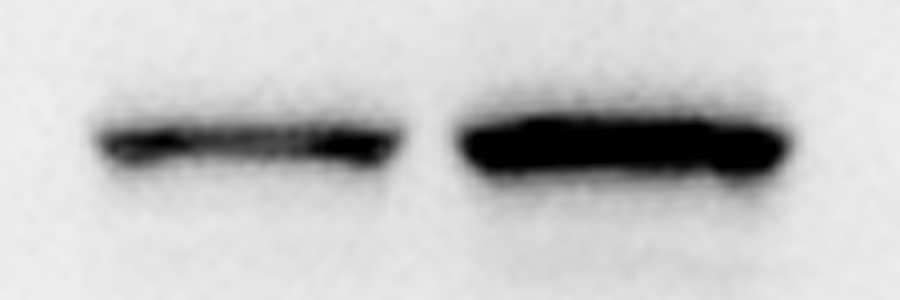

Supplement: Supplemental Information 1 [file peerj-12-16818-s001.zip › Raw data/WB/Trem2-25kd.tif]

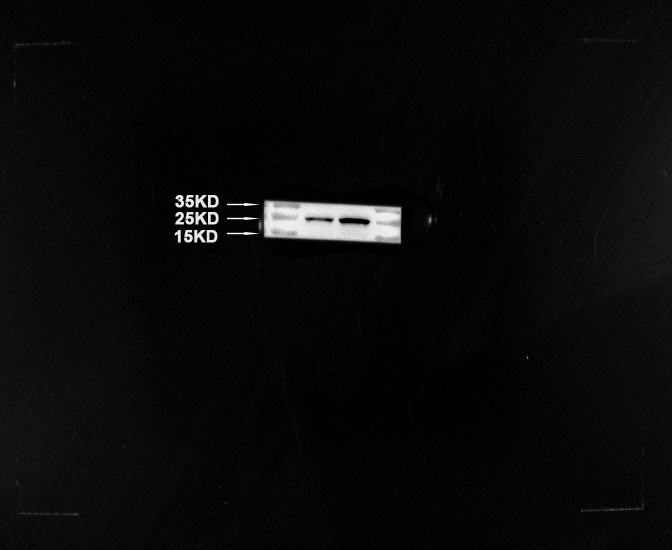

Supplement: Supplemental Information 1 [file peerj-12-16818-s001.zip › Raw data/WB/Trem2-Marker.tif]
