# Supplementary material for: Study on pyroptosis-related genes Casp8, Gsdmd and Trem2 in mice with cerebral infarction
Source: PeerJ. 2024 Feb 9;12:e16818. doi: 10.7717/peerj.16818 (PMC10860548; doi:10.7717/peerj.16818)
Supplement: Supplemental Information 2 [file peerj-12-16818-s002.doc]

**Attached table 1**

| GO: 0070269 Dataset Genes |
| --- |
| Symbol |
| Aim2 |
| Apip |
| Casp1 |
| Casp4 |
| Casp8 |
| Dhx9 |
| Gsdma |
| Gsdma2 |
| Gsdma3 |
| Gsdmc |
| Gsdmc2 |
| Gsdmc3 |
| Gsdmc4 |
| Gsdmd |
| Gsdme |
| Gzma |
| Gzmb |
| Gzmc |
| Gzmd |
| Gzme |
| Gzmf |
| Gzmg |
| Gzmn |
| Naip1 |
| Naip2 |
| Naip5 |
| Naip6 |
| Naip7 |
| Nlrc4 |
| Nlrp1a |
| Nlrp1b |
| Nlrp6 |
| Nlrp9b |
| Trem2 |
| Zbp1 |
