# Supplementary material for: Study on pyroptosis-related genes Casp8, Gsdmd and Trem2 in mice with cerebral infarction
Source: PeerJ. 2024 Feb 9;12:e16818. doi: 10.7717/peerj.16818 (PMC10860548; doi:10.7717/peerj.16818)
Supplement: Supplemental Information 4 [file peerj-12-16818-s004.doc]

**MIQE Guidelines for qPCR**

**Special Report**

| **Table 1. MIQE checklist for authors, reviewers, and editors.a** |
| --- |
| **Item to check Importance Item to check Importance** |
| Experimental design qPCR oligonucleotides |
| Definition of experimental and control groups E √ Primer sequences E |
| Number within each group E √ RTPrimerDB identification number D |
| Assay carried out by the core or investigator’s laboratory? D Probe sequences Dd |
| Acknowledgment of authors’ contributions D Location and identity of any modifications E √ |
| Sample Manufacturer of oligonucleotides D |
| Description E √ Purification method D |
| Volume/mass of sample processed D qPCR protocol |
| Microdissection or macrodissection E √ Complete reaction conditions E √ |
| Processing procedure E √ Reaction volume and amount of cDNA/DNA E √ |
| If frozen, how and how quickly? E √ Primer, (probe), Mg^2+^, and dNTP concentrations E √ |
| If fixed, with what and how quickly? E √ Polymerase identity and concentration E √ |
| Sample storage conditions and duration (especially for FFPEb samples) E √ Buffer/kit identity and manufacturer E √ |
| Nucleic acid extraction Exact chemical composition of the buffer D |
| Procedure and/or instrumentation E √ Additives (SYBR Green I, DMSO, and so forth) E √ |
| Name of kit and details of any modifications E √ Manufacturer of plates/tubes and catalog number D |
| Source of additional reagents used D Complete thermocycling parameters E √ |
| Details of DNase or RNase treatment E √ Reaction setup (manual/robotic) D |
| Contamination assessment (DNA or RNA) E √ Manufacturer of qPCR instrument E √ |
| Nucleic acid quantification E √ qPCR validation |
| Instrument and method E √ Evidence of optimization (from gradients) D |
| Purity (A260/A280) D Specificity (gel, sequence, melt, or digest) E √ |
| Yield D For SYBR Green I, Cq of the NTC E √ |
| RNA integrity: method/instrument E √ Calibration curves with slope and y intercept E √ |
| RIN/RQI or Cq of 3, and 5, transcripts E √ PCR efficiency calculated from slope E √ |
| Electrophoresis traces D CIs for PCR efficiency or SE D |
| Inhibition testing (Cq dilutions, spike, or other) E √ r2 of calibration curve E √ |
| Reverse transcription Linear dynamic range E √ |
| Complete reaction conditions E √ Cq variation at LOD E √ |
| Amount of RNA and reaction volume E √ CIs throughout range D |
| Priming oligonucleotide (if using GSP) and concentration E √ Evidence for LOD E √ |
| Reverse transcriptase and concentration E √ If multiplex, efficiency and LOD of each assay E √ |
| Temperature and time E √ Data analysis |
| Manufacturer of reagents and catalogue numbers D qPCR analysis program (source, version) E √ |
| Cqs with and without reverse transcription Dc Method of Cq determination E √ |
| Storage conditions of cDNA D Outlier identification and disposition E √ |
| qPCR target information Results for NTCs E √ |
| Gene symbol E √ Justification of number and choice of reference genes E √ |
| Sequence accession number E √ Description of normalization method E √ |
| Location of amplicon D Number and concordance of biological replicates D |
| Amplicon length E √ Number and stage (reverse transcription or qPCR) of technical replicates E √ |
| In silico specificity screen (BLAST, and so on) E √ Repeatability (intraassay variation) E √ |
| Pseudogenes, retropseudogenes, or other homologs? D Reproducibility (interassay variation, CV) D |
| Sequence alignment D Power analysis D |
| Secondary structure analysis of amplicon D Statistical methods for results significance E √ |
| Location of each primer by exon or intron (if applicable) E √ Software (source, version) E √ |
| What splice variants are targeted? E √ Cq or raw data submission with RDML D |
| a All essential information (E) must be submitted with the manuscript. Desirable information (D) should be submitted if available. If primers are from RTPrimerDB, information on qPCR target, oligonucleotides, protocols, and validation is available from that source.  b FFPE, formalin-fixed, paraffin-embedded; RIN, RNA integrity number; RQI, RNA quality indicator; GSP, gene-specific priming; dNTP, deoxynucleoside triphosphate.  c Assessing the absence of DNA with a no–reverse transcription assay is essential when first extracting RNA. Once the sample has been validated as DNA free, inclusion of a no–reverse transcription control is desirable but no longer essential.  d Disclosure of the probe sequence is highly desirable and strongly encouraged; however, because not all vendors of commercial predesigned assays provide this  information, it cannot be an essential requirement. Use of such assays is discouraged. |
